# Inbred varieties outperformed hybrid rice varieties under dense planting with reducing nitrogen

**DOI:** 10.1038/s41598-020-65574-0

**Published:** 2020-05-29

**Authors:** Jian Lu, Danying Wang, Ke Liu, Guang Chu, Liying Huang, Xiaohai Tian, Yunbo Zhang

**Affiliations:** 1 Hubei Collaborative Innovation Center for Grain Industry, Yangtze University, 434025 Jingzhou, China; 2 China National Rice Research Institute, 310006 Hangzhou, China; 3 College of Agriculture, Yangtze University, 434025 Jingzhou, China; 4 Engineering Research Center of Ecology and Agricultural Use of Wetland, Ministry of Education, Jingzhou, 434025 China

**Keywords:** Plant ecology, Plant physiology

## Abstract

Field experiments were conducted over two years to evaluate the effects of planting density and nitrogen input rate on grain yield and nitrogen use efficiency (NUE) of inbred and hybrid rice varieties. A significant interaction effect was observed between nitrogen input and planting density on grain yield. Higher number of panicles per square meter and spikelets per panicle largely accounted for the observed advantage in performance of inbred, relative to hybrid varieties. Compared with high nitrogen input rate, nitrogen absorption efficiency, nitrogen recovery efficiency, and partial factor productivity increased by 24.6%, 28.0%, and 33.3% in inbred varieties, and by 32.2%, 29.3%, and 35.0% in hybrids under low nitrogen input, respectively. Inbred varieties showed higher nitrogen absorption efficiency, nitrogen recovery efficiency, and partial factor productivity than hybrids, regardless of nitrogen input level. Nitrogen correlated positively with panicle number, spikelets per panicle, biomass production at flowering, and after flowering in inbred varieties but only with panicle number and biomass production at flowering in hybrids. Inbred varieties are more suitable for high planting density at reduced nitrogen input regarding higher grain yield and NUE. These findings bear important implications for achieving high yield and high efficiency in nutrient uptake and utilization in modern rice-production systems.

## Introduction

Rice (*Oryza sativa* L.) is the main staple food for more than half the population of the world^1^. As one of the largest rice producers and consumers, China occupied 18.8% of the global rice-growing area and accounted for 28.1% of the total production in 2014^2^. Double-season rice cropping has significantly contributed to the increase in rice productivity in China; however, the cultivated area has decreased substantially due to labor migration and increased labor costs over the past decades^3^. Therefore, it is necessary to develop labor-saving cultivation technologies to reverse the declining trend in rice cultivation area in this country.

Mechanical transplanting is an alternative labor-saving technology in rice production^4^. As efficient agriculture has been popularized in recent years, mechanical transplanting has been rapidly adopted for rice production in China^5^. However, rice farmers still follow traditional field management practices even under the mechanical-transplanting production scheme^6^. Previous studies confirmed that certain traditional management practices, such as nitrogen (N) fertilization utilize resources inefficiently and have negative environmental impacts^7,8^. In China, the average N application rate for rice production is 180 kg ha^−1^, which is 75% higher than the global average^9–11^. Consequently, only 20–30% of applied N is actually absorbed by the crop, while most of it is lost to the environment^6^. Over the past three decades, this N-fertilizer overuse in China has caused surface water eutrophication, soil acidification, increased greenhouse gas emissions, and enhanced N deposition^9–14^. Moreover, diminishing returns are being observed with N fertilizer use in China; indeed, the resulting increases in rice production have not been commensurate with the increases in N fertilizer application since the start of the Green Revolution^15,16^. Therefore, external N input must be reduced in order to realize both environmental and economic benefits from rice production.

High planting density has been recommended to reduce N application rates in rice production^15^. Thus, for example, Liu *et al*.^17^ demonstrated that for conventional seedling broadcasting, the N application rate was lowered by 18% when seedling density was raised by 32%; nevertheless, grain yield was not substantially improved. On the other hand, Hou *et al*.^18^ observed that a 165 kg N ha^−1^ application rate combined with a 24–27 × 10^4^ hills ha^−1^ for planting density resulted in similar or even greater grain yield and NUE than with a 245.5 kg N ha^−1^ application rate on mechanically transplanted hybrid rice. Similar results were reported by Huang *et al*.^15^ and Xie *et al*.^19^, who claimed that high planting density, combined with reduced N input rate, may increase grain yield and NUE even under low light stress. The aforementioned results indicate that high-density planting with lowered N input might be a sustainable strategy for the improvement of rice yield and NUE.

Most previous studies focused on only one type of rice variety. In contrast, very few studies have compared the relative effects of combining high planting density with reduced nitrogen application rate on yield and NUE in inbred and hybrid varieties simultaneously. Super hybrid cultivars have high yield potential associated with a relatively larger number of spikelets per panicle and higher biomass production than inbred rice cultivars^20–22^. Thus, for example, Zhang *et al*.^20^ observed that super hybrid varieties had 12% higher yield potential than ordinary hybrid and inbred varieties. They hypothesized that mechanically transplanted hybrid-rice cultivated at low N input and high planting density, may still show superior grain yield compared with inbred varieties.

In this study, field experiments were conducted using inbred and hybrid varieties grown at different N application rates and planting densities. We used the method of cluster analysis, and the different agronomic characters of inbred and hybrid rice were analyzed by variance analysis, and the effect of nitrogen rate and planting density of the same type of rice was compared further. The objectives of this study were: 1) to compare grain yield and NUE between inbred and hybrid rice varieties grown under different N input rates and planting densities, and 2) to identify the factors accounting for the relative differences in grain yield and NUE between inbred and hybrid rice varieties.

## Results

### Climate conditions

**Figure 1:**
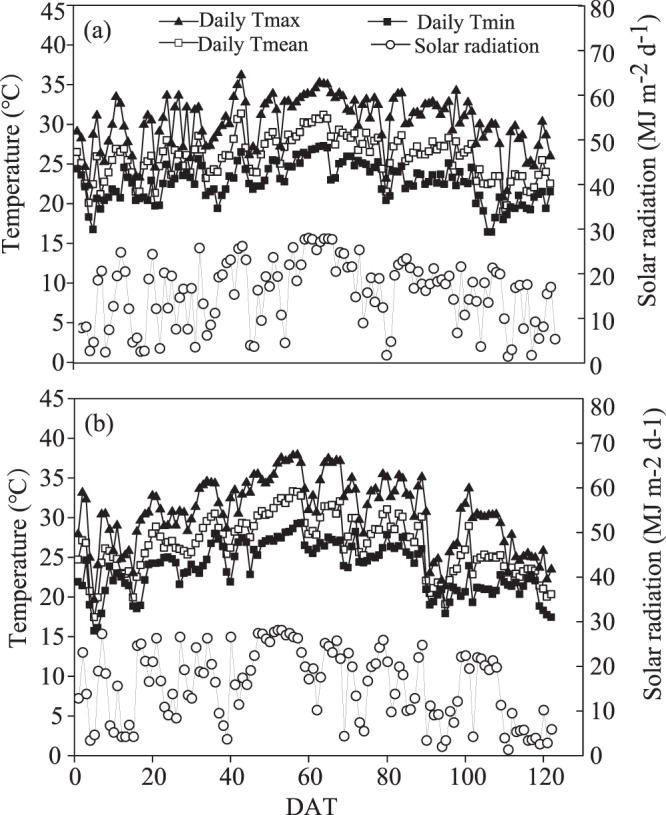
Daily maximum and minimum temperatures and solar radiation during the rice-growing season at Jingzhou in 2017 (**a**) and Hangzhou in 2018 (**b**).

Average temperature during the growing season was 0.5–1.8 °C lower in Jingzhou than in Hangzhou (Fig. 1). Seasonal mean maximum temperatures in Jingzhou were 30.0 °C in 2017 and 32.4 °C in Hangzhou in 2018. Seasonal mean minimum temperatures in Jingzhou were 23.0 °C in 2017 and 24.2 °C in Hangzhou in 2018, respectively. As can be seen, temperatures did not differ much between Jingzhou and Hangzhou. Further, the difference in average daily solar radiation during the growing season was only ~7.3% between the two sites. The seasonal average daily radiation levels were 15.9 MJ m^−2^ d^−1^ and 14.7 MJ m^−2^ d^−1^ in 2017 and 2018 for Jingzhou and Hangzhou, respectively. Therefore, the difference in seasonal average daily radiation between Jingzhou and Hangzhou was similar.

### Grain yield

N application rate, planting density, and variety significantly (*P* < 0.01) affected grain yield of hybrid and inbred cultivars at both sites (Tables 1 and 2). However, the interactive effects of these factors on grain yield were not significant (*P* > 0.05). Grain yield significantly increased (N1) and either remained constant (N2) or decreased (N3) with increasing N application rate at all planting densities. Yield increase in inbred and hybrid varieties differed among N treatments. Average grain-yield increases were 26.32% for the inbred varieties and 8.84% for the hybrid varieties (Tables 1 and 2). At Jingzhou, N1×D3 was the most effective of all combination treatments to increase grain yield in both inbred and hybrid varieties. In turn, at Hangzhou, N3×D3 realized the highest grain yield for the inbred varieties, while N3×D1 was optimal for enhancing grain yield in the hybrids tested. Varietal differences were not significant (*P* > 0.05) across N treatments, except for N0 at Jingzhou and N3×D2 at Hangzhou.

**Table 1:** Grain yield (kg ha^−1^) relative N application rate and planting density in inbred and hybrid rice at Jingzhou in 2017.

Variety	N0			N1			N2			N3		
	D1	D2	D3	D1	D2	D3	D1	D2	D3	D1	D2	D3
HHZ	4.35b	4.55b	5.36b	8.56b	9.60a	10.46a	10.25a	9.79a	9.59a	10.35ab	9.69a	9.14b
YNSM	4.65b	4.57b	5.28b	9.59a	9.91a	10.45a	10.30a	9.75a	9.60a	10.44a	9.70a	9.48b
Y-LY900	7.73a	8.47a	8.67a	9.77a	10.13a	10.43a	9.53b	10.03a	10.13a	9.90a	9.60a	10.43a
QLYSM	8.01a	7.90a	8.73a	9.53a	9.67a	10.43a	8.77c	8.80b	9.73a	9.07b	8.73b	9.63b
ANOVA	Variety (V)	**										
	Nitrogen (N)	**										
	Density (D)	*										
	V*N	**										
	V*D	ns										
	N*D	ns										
	V*N*D	ns										

† Different lowercase letters within columns indicate significant differences among varieties at P < 0.05 (n = 3). *P < 0.05. **P < 0.01. ns, not significant.

**Table 2:** Grain yield (kg ha^−1^) relative N application rate and planting density in inbred and hybrid rice at Hangzhou in 2018.

Variety	N0			N1			N2			N3		
	D1	D2	D3	D1	D2	D3	D1	D2	D3	D1	D2	D3
HHZ	6.24a	6.24bc	7.04a	8.12a	8.78b	9.34ab	9.80a	9.61a	9.33ab	10.11a	10.27a	11.32a
YD-6	5.43a	5.91c	6.55a	6.72b	7.74c	8.21ac	8.62b	8.61b	8.90b	7.92b	8.54b	9.92ab
ZZY-8	6.57a	6.82ab	7.11a	8.47a	9.72a	8.41bc	9.31a	9.29ab	9.81ab	9.22a	9.12b	9.31b
C-LYHZ	6.90a	7.21a	7.02a	8.79a	9.22ab	9.52a	9.91a	9.13ab	9.81a	10.22a	10.23a	10.04ab
ANOVA	Variety (V)		**									
	Nitrogen (N)		**									
	Density (D)		**									
	V*N		ns									
	V*D		ns									
	N*D		ns									
	V*N*D		ns									

^†^Different lowercase letters within columns indicate significant differences among varieties at P < 0.05 (n = 3). *P < 0.05. **P < 0.01. ns, not significant.

### Yield components, biomass, and NUE

Yield component responses to plant density varied with N application rate (Tables 3 and 4). Panicle number significantly (*P* < 0.05) increased with N application rate and plant density for inbred and hybrid rice varieties. The number of spikelets per panicle significantly (*P* < 0.05) increased with N application rate but decreased with increasing planting density. The number of panicles in the treatments receiving N was 22.1% and 32.1% higher for the hybrid and the inbred varieties, respectively, relative to the N0 treatment, (Tables 3 and 4). Conversely, relative to N0, the number of spikelets per panicle in all N treatments was 27.0% and 9.9%, respectively, higher for the inbred and the hybrid varieties (Tables 3 and 4). At Jingzhou, N1×D3 was optimal for inducing greater panicle and spikelet number per panicle across treatments in both inbred and hybrid varieties (Table 3). In turn, at Hangzhou, the optimal combination treatment for promoting higher panicle and spikelets number per panicle in inbred varieties was N3×D3, while N3×D1 was the best combination treatment for the hybrid varieties under study (Table 4

**Table 3:** Yield components relative to N application rate and planting density in inbred and hybrid rice varieties at Jingzhou. Different lowercase letters indicate significant differences at P < 0.05 (n = 3).

Variety	N	D	Panicles	Skikete panicle^−1^	Seed setting precentage(%)	1000-grain weight(mg)
**HHZ**	N0	D1	214.5d	118.5d	90.3a	20.2a
		D2	231.8 cd	104.5d	90.5a	20.2a
		D3	262.9c	128.7d	89.4ab	19.8ab
	N1	D1	345.1b	167.8c	89.5ab	19.5abc
		D2	354.2ab	188.6abc	88.4abc	19.5abc
		D3	397.2a	196.5abc	83.5abcd	18.9cde
	N2	D1	354.7ab	198.5abc	87.6abc	19.3bc
		D2	374.6ab	180.5bc	85.2abcd	18.6def
		D3	396.3a	180.8bc	81.4bcd	18.2f
	N3	D1	376.2ab	215.2a	80.1cd	19.0cd
		D2	390.4ab	199.8ab	78.2d	18.2ef
		D3	395.6a	190.4abc	78.7d	18.0f
**YNSM**	N0	D1	240.3a	123.5b	90.8a	20.4a
		D2	239.5c	117.9b	90.3a	20.2ab
		D3	261.0c	123.5b	90.2a	20.2ab
	N1	D1	332.8d	197.5a	87.4abc	20.1abc
		D2	342.7cd	189.8a	84.6abcd	19.8abc
		D3	394.4abc	192.6a	84.3abcd	19.5abcd
	N2	D1	353.3bcd	210.5a	87.8ab	19.5abcd
		D2	371.2abcd	191.5a	84.5abcd	19.1abcd
		D3	401.6ab	195.5a	81.8bcd	18.8bcd
	N3	D1	377.1abcd	219.2a	81.5bcd	18.8cd
		D2	390.6abc	201.2a	80.7cd	18.4cd
		D3	409.3a	190.6a	78.5d	18.3d
**Y-LY900**	N0	D1	151.6cef	339.1ab	79.0ab	20.7b
		D2	147.3cf	319.7ab	80.8ab	21.1ab
		D3	212.0abc	221.5 cd	83.5a	21.6ab
	N1	D1	198.3bcd	260.4abcd	61.3e	22.2a
		D2	200.8bc	254.3bcd	72.3bcd	20.8b
		D3	242.1ab	209.2d	70.9bcde	21.5ab
	N2	D1	168.8cdef	344.4a	63.0de	21.9ab
		D2	187.5cde	338.6ab	66.5de	21.5ab
		D3	257.3a	295.0abcd	68.1cde	21.8ab
	N3	D1	132.9f	266.6abcd	72.5bcd	21.1ab
		D2	176.6cdef	304.9abc	67.1de	21.3ab
		D3	173.9cdef	302.6abc	77.8abc	21.4ab
**QLYSM**	N0	D1	177.8cd	167.5bcde	87.4ab	24.2cd
		D2	171.7d	217.1ab	72.3de	24.6abc
		D3	210.7bcd	133.5e	89.7a	24.3bcd
	N1	D1	179.2 cd	246.6a	83.9abc	24.3abcd
		D2	218.9bcd	208.3abc	75.0cde	24.7ab
		D3	257.6b	188.6abcde	82.3abcd	24.5abc
	N2	D1	164.0d	221.0ab	70.3e	24.2cd
		D2	262.5b	128.6e	75.6cde	24.3bcd
		D3	268.8b	142.2de	80.5abcde	24.0d
	N3	D1	244.9bc	151.6cde	77.5bcde	24.8a
		D2	247.5b	194.7abcd	78.9bcde	24.7ab
		D3	432.0a	130.8e	82.2abcd	24.5abc
ANOVA		Variety (V)	ns	ns	ns	*
		Nitrogen (N)	**	**	ns	ns
		Density (D)	**	*	ns	ns
		V*N	*	ns	*	*
		V*D	ns	ns	ns	ns
		N*D	ns	ns	ns	ns
		V*N*D	*	ns	ns	ns

^†^Different lowercase letters within columns indicate significant differences at P < 0.05 across N rates (n = 3).

**Table 4:** Yield components relative to N application rate and planting density in inbred and hybrid rice varieties at Hangzhou.

Variety	N	D	Panicles	Skikete panicle^−1^	Seed setting precentage(%)	1000-grain weight(mg)
**HHZ**	N0	D1	196.8g	165.5b	92.8a	18.9ab
		D2	223.5fg	169.8ab	91.9a	18.8ab
		D3	244.7defg	166.8b	92.7a	19.0ab
	N1	D1	240.5efg	170.1ab	92.7a	18.8ab
		D2	259.5cdef	177.3ab	92.4a	19.0ab
		D3	304.0bc	174.0ab	93.2a	18.1b
	N2	D1	278.5cde	191.0a	91.5a	18.2b
		D2	348.6ab	184.7ab	91.2a	19.0ab
		D3	377.2a	186.3ab	94.3a	18.5ab
	N3	D1	358.3a	190.9a	91.0a	19.1ab
		D2	297.4bcd	190.7a	92.6a	18.9ab
		D3	367.9a	182.8ab	93.2a	19.4a
**YD-6**	N0	D1	161.0bc	147.6bcd	91.9a	28.1ab
		D2	177.5bc	135.5d	92.4a	27.9b
		D3	142.7c	143.3cd	89.9ab	28.3ab
	N1	D1	169.9bc	188.7a	91.3a	28.2ab
		D2	217.0ab	182.3ab	90.3ab	28.3ab
		D3	172.9bc	159.3abcd	87.5ab	28.5ab
	N2	D1	173.1bc	193.2a	92.2a	28.2ab
		D2	221.7ab	174.9abc	89.5ab	28.2ab
		D3	211.9ab	177.0abc	83.5b	28.5ab
	N3	D1	205.8abc	189.2a	91.0a	27.9b
		D2	248.3a	177.8abc	90.0ab	28.9a
		D3	265.9a	166.7abcd	85.4ab	28.0b
**ZZY-8**	N0	D1	192.6d	178.7c	83.3abc	22.5a
		D2	203.6 cd	193.8bc	86.6a	22.6a
		D3	212.1bcd	200.1bc	83.4ab	22.6a
	N1	D1	226.6abcd	233.8ab	81.5abcd	22.8a
		D2	247.3abc	230.1ab	80.3abcd	22.6a
		D3	233.9abcd	237.7ab	81.0abcd	22.1a
	N2	D1	236.3abcd	250.9a	80.1abcd	22.5a
		D2	242.2abcd	246.1a	78.4bcde	22.7a
		D3	262.5ab	245.2a	81.4abcd	22.5a
	N3	D1	246.8abc	249.1a	75.4de	22.6a
		D2	264.8ab	247.5a	70.9e	22.8a
		D3	271.5a	251.1a	75.7cde	22.6a
**C-LYHZ**	N0	D1	201.2e	183.3b	86.3a	20.9a
		D2	235.1 cde	181.9b	84.7ab	20.7ab
		D3	226.5de	179.6b	84.5ab	20.9a
	N1	D1	292.0ab	211.8ab	79.0abc	20.4ab
		D2	308.7ab	214.8ab	78.2abc	20.4ab
		D3	285.8abc	212.5ab	80.2abc	21.1a
	N2	D1	316.0ab	218.6ab	79.1abc	21.0a
		D2	273.9bcd	227.6a	74.9c	19.9b
		D3	304.5ab	225.4a	74.3c	20.4ab
	N3	D1	333.6a	226.2a	77.0bc	20.7ab
		D2	323.9ab	230.1a	73.4c	20.5ab
		D3	337.8a	231.5a	73.7c	20.9a
ANOVA		Variety (V)	ns	ns	ns	ns
		Nitrogen (N)	**	*	ns	*
		Density (D)	*	ns	ns	ns
		V*N	*	**	ns	ns
		V*D	ns	ns	ns	ns
		N*D	*	ns	ns	ns
		V*N*D	ns	ns	ns	ns

^†^Different lowercase letters within columns indicate significant differences at P < 0.05 across N rates (n = 3).

Different lowercase letters indicate significant differences at P < 0.05 (n = 3).

).

The effects of N application rate and plant density on percent seed set were not significant (*P* > 0.05) for the inbred varieties but they were (*P* < 0.05) for the hybrid varieties. In contrast, grain filling significantly (*P* < 0.05) decreased with increasing N application rate in hybrid rice varieties. Grain filling in ZZY-8 and C-LYHZ decreased from 85% under N0 to 75% under N3. 1,000-grain weight did not (*P* > 0.05) change significantly in either rice varietal group across N application rates or planting densities. Interaction analysis showed that N application rate significantly (*P* < 0.05) influenced panicle number, spikelet number per panicle, percent grain filling, and 1,000-grain weight, whereas planting densities only (*P* < 0.05) affected significantly panicle number and spikelet number per panicle.

### Biomass, nitrogen uptake, and NUE

Total aboveground biomass at maturity significantly (*P* < 0.05) increased with N application rate and planting density (Tables 5 and 6). At both sites and across N treatments, ordinary hybrids had consistently higher biomass than inbred varieties. Total aboveground biomass for hybrid varieties was 14.3% higher than that for inbred varieties (Tables 5 and 6). Total aboveground biomass at maturity under all N treatments was 10.1% and 33.7% higher in hybrids and inbred varieties compared with the N0 treatment, respectively (Tables 5 and 6

**Table 5:** Biomass at maturity, nitrogen uptake, and NUE relative to N application rate and planting density in inbred and hybrid rice at Jingzhou in 2017.

Variety	N	D	Biomass	AE	RE	PFP
			(g m^−2^)	(kg kg^−1^)	(%)	(kg kg^−1^)
**HHZ**	N0	D1	764.7d			
		D2	823.2d			
		D3	873.6d			
	N1	D1	1125.0c	31.2c	26.7bc	63.4c
		D2	1294.5abc	37.5ab	34.9a	71.1b
		D3	1362.6ab	37.8a	36.2a	77.5a
	N2	D1	1250.3bc	32.8bc	27.0bc	57.0d
		D2	1293.5abc	29.1cd	26.1bc	54.4d
		D3	1370.4ab	23.5ef	27.6b	53.3d
	N3	D1	1227.1bc	28.6 cd	20.6d	46.0e
		D2	1335.7abc	24.5de	22.8 cd	43.1ef
		D3	1485.3a	18.8 f	27.2bc	40.7 f
**YNSM**	N0	D1	810.3d			
		D2	830.2d			
		D3	870.2d			
	N1	D1	1290.3bc	36.6a	35.6bc	71.6b
		D2	1341.0abc	39.6a	37.9b	73.5b
		D3	1480.5ab	38.3a	45.2a	77.4a
	N2	D1	1204.5c	31.4b	21.9e	57.3c
		D2	1337.4abc	28.8bc	28.2d	54.2 cd
		D3	1486.6ab	24.0d	34.3c	53.3d
	N3	D1	1352.5abc	25.8cd	24.1e	46.4e
		D2	1484.4ab	22.8de	29.1d	43.1ef
		D3	1541.9a	18.6e	29.9d	42.1 f
**Y-LY900**	N0	D1	1216.1de			
		D2	1518.2ab			
		D3	1533.0a			
	N1	D1	1248.8d	8.0b	17.1cd	54.3a
		D2	1580.5a	9.3a	39.2a	56.3a
		D3	1531.1a	9.4a	34.5b	58.0a
	N2	D1	1264.3d	6.5d	8.2e	45.1b
		D2	1437.0c	7.0c	30.7b	44.6b
		D3	1557.4a	5.0e	32.8b	42.4bc
	N3	D1	1157.5e	4.3f	15.2d	37.4cd
		D2	1450.2bc	4.2f	20.7c	35.6d
		D3	1527.5a	4.1f	20.5c	34.2d
**QLYSM**	N0	D1	1199.2 g			
		D2	1429.8bcd			
		D3	1491.3ab			
	N1	D1	1371.5de	10.3ab	61.7a	58.0a
		D2	1483.9abc	9.8b	38.6b	53.7b
		D3	1468.8abc	13.0a	15.6c	53.0b
	N2	D1	1252.3fg	4.9cd	15.9c	43.3c
		D2	1413.3 cd	4.0d	19.5c	39.1d
		D3	1538.9a	7.4bc	4.8d	39.0d
	N3	D1	1204.4 g	5.2cd	34.3b	35.7e
		D2	1309.4ef	3.1d	18.4c	32.4e
		D3	1479.4abc	5.8cd	2.4d	33.6e
ANOVA		Variety (V)	**	*	ns	ns
		Nitrogen (N)	**	**	*	*
		Density (D)	*	*	ns	ns
		V*N	**	ns	*	*
		V*D	ns	ns	ns	ns
		N*D	ns	ns	ns	ns
		V*N*D	**	ns	ns	ns

^†^Different lowercase letters within columns indicate significant differences at P < 0.05 across N rates (n = 3).

**Table 6:** Biomass at maturity, nitrogen uptake, and NUE relative to N application rate and planting density in inbred and hybrid rice at Hangzhou in 2018.

Variety	N	D	Biomass	AE	RE	PFP
			(g m^−2^)	(kg kg^−1^)	(%)	(kg kg^−1^)
HHZ	N0	D1	781.2f			
		D2	1007.4ef			
		D3	1134.6e			
	N1	D1	1393.5d	15.7f	32.6b	67.6c
		D2	1580.4 cd	21.3a	31.5bc	72.8b
		D3	1827.4abc	19.6bcd	39.3a	77.7a
	N2	D1	1459.8d	18.8d	25.4e	56.5de
		D2	1458.1d	21.0ab	22.0f	58.4d
		D3	1976.8a	17.1ef	28.9 cd	59.3d
	N3	D1	1593.7bcd	18.5de	27.1de	48.1 f
		D2	1611.5bcd	19.3cd	17.3g	48.7 f
		D3	1831.3ab	20.6abc	18.7g	53.8e
YD-6	N0	D1	1076.5bcd			
		D2	1112.5bcd			
		D3	1020.7cd			
	N1	D1	1031.8 cd	10.7f	14.9c	55.7b
		D2	1340.1ab	14.6bc	17.1b	64.1a
		D3	1613.7a	13.5cde	14.5c	67.9a
	N2	D1	898.4d	19.3a	14.4c	52.0b
		D2	1156.8bcd	15.9b	19.8a	51.9bc
		D3	1240.2bc	14.1bcd	11.3d	53.7b
	N3	D1	1041.1cd	11.7ef	11.6d	37.4d
		D2	1350.8ab	12.4def	11.7d	40.7d
		D3	1586.9a	15.9b	14.8c	47.0c
ZZY-8	N0	D1	1093.9e			
		D2	1077.0e			
		D3	1761.6abc			
	N1	D1	1237.9de	15.5bc	10.2g	70.0b
		D2	1640.5abc	23.7a	20.2b	80.5a
		D3	1936.0a	10.6e	32.9a	70.1b
	N2	D1	1383.5cde	17.0b	15.3de	56.7c
		D2	1671.4abc	14.7c	18.7c	56.0c
		D3	1588.7abcd	16.4bc	16.2d	59.7c
	N3	D1	1532.7bcd	12.8d	15.0e	43.9d
		D2	1908.0ab	10.7e	19.9b	43.1d
		D3	1854.1ab	10.2e	11.9f	44.2d
C-LYHZ	N0	D1	1155.5f			
		D2	1373.5def			
		D3	1727.7bc			
	N1	D1	1271.9ef	14.2d	15.3d	72.1c
		D2	1595.6 cd	16.3bcd	20.2b	76.2b
		D3	1915.3ab	21.1a	14.6d	79.3a
	N2	D1	1271.4ef	18.2b	14.0d	60.3d
		D2	1663.1c	11.7e	18.0c	55.3e
		D3	2025.2a	17.1bc	20.8b	59.4d
	N3	D1	1507.7cde	15.5 cd	17.5c	48.5f
		D2	1944.6ab	14.8d	25.3a	49.1f
		D3	2098.2a	14.5d	17.3c	47.8f
ANOVA		Variety (V)	**	*	ns	ns
		Nitrogen (N)	**	**	*	*
		Density (D)	*	*	ns	ns
		V*N	**	ns	*	*
		V*D	ns	ns	ns	ns
		N*D	ns	ns	ns	ns
		V*N*D	**	ns	ns	ns

^†^Different lowercase letters within columns indicate significant differences at P < 0.05 across N rates (n = 3).

). At Jingzhou, N2×D3 was the optimal combination treatment for higher biomass accumulation in both inbred and hybrid varieties. In turn, at Hangzhou, N3×D3 was optimal for elevated biomass accumulation in inbred varieties and N3×D1 was best for increased biomass accumulation in hybrid varieties.

Nitrogen uptake and NUE varied among treatments (Tables 5 and 6). N uptake and NUE under the various N application rates and planting densities were similar at both sites. Inbred varieties showed higher AE, RE, and PFP than hybrids. AE and PFP significantly (*P* < 0.05) increased with planting density at low N rates (N1) but significantly (*P* < 0.05) decreased with increasing planting density at high N rates (N2 and N3) for both types of variety. RE increased with planting density but decreased with N application rate. AE, RE, and PFP were 24.6%, 28.0%, and 33.3% higher in inbred varieties and 32.2%, 29.3%, and 35.0% higher in hybrid varieties under the N1 treatment relative to N3 (Tables 5 and 6). At Jingzhou, N1×D3 was optimal for higher AE, RE, and PFP for both inbred and hybrid varieties. In turn, at Hangzhou, N1×D3 was optimal for higher AE, RE, and PFP in hybrid varieties, while N1×D2 was optimal for higher AE, RE, and PFP in inbred varieties.

### Correlation analyses of grain yield, yield components, and biomass at various nitrogen application rates and planting densities

**Figure 2:**
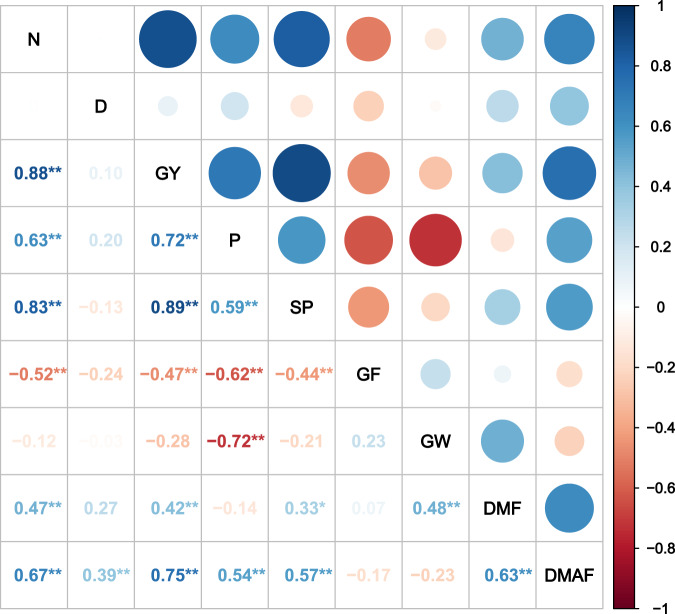
Correlation matrix of various grain yield parameters and biomass in inbred rice (n = 42). N, nitrogen application rate; D, planting density; P, panicles m^-2^; S, spikelets per panicle; GF, percent seed set; GW, 1,000-grain weight; DMF, dry matter production at flowering; DMAF, dry matter production after flowering; Y, grain yield. Numbers are determination coefficients. *P < 0.05. **P < 0.01.

**Figure 3:**
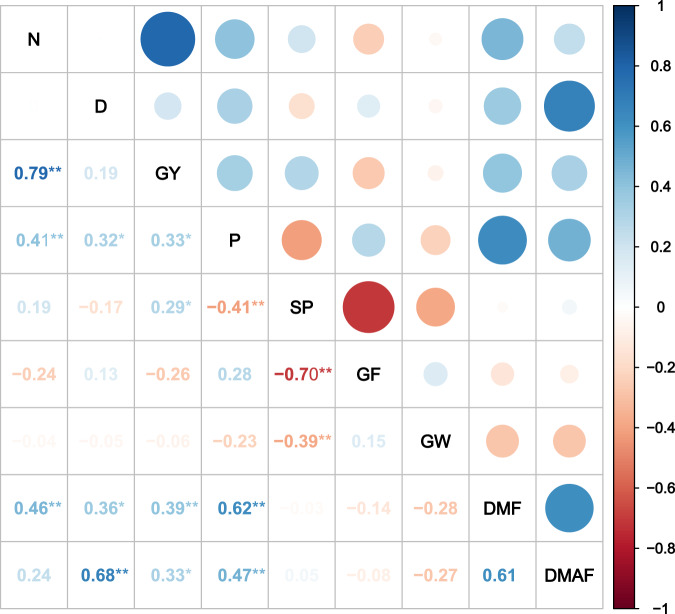
Correlation matrix of various grain yield parameters and biomass in hybrid rice (n = 48). N, nitrogen application rate; D, planting densities; P, panicles m^-2^; S, spikelets per panicle; GF, percent seed set; GW, 1,000-grain weight; DMF, dry matter production at flowering; DMAF, dry matter production after flowering; Y, grain yield. Numbers are determination coefficients. *P < 0.05. **P < 0.01.

Correlation matrices among the various grain yield components and biomass parameters for inbred and hybrid varieties are shown in Figs. 2 and 3. N application rate significantly (*P* < 0.01) and positively correlated with GY, P, SP, DMF, and DMAF for the inbred varieties and with GY, P, and DMF for the hybrid varieties. Additionally, there was a significant (*P* < 0.01) positive correlation between D and DMAF for both the inbred and hybrid varieties. The significant (*P* < 0.01) positive correlations among GY, P, SP, DMF, and DMAF were identical for the inbred and hybrid varieties. However, GF was significantly (*P* < 0.05) and negatively correlated with GY for both inbred and hybrid varieties.

## Discussion

Several studies have confirmed that super hybrid rice varieties had higher yields than ordinary hybrids or inbred varieties^20,23,24^. However, it is not conclusively known whether the same holds under conditions of high planting density and reduced nitrogen application rate. The present study compared yield and NUE for inbred and hybrid rice varieties cultivated under different N rates and planting densities. Neither super nor ordinary hybrid varieties surpassed inbred varieties in terms of grain yield or NUE.

Neither the super hybrid rice variety (Y-LY900) nor the ordinary hybrid rice varieties (ZZY-8, C-LYHZ and QLYSM) showed any significant advantage over the inbred varieties (HHZ and YNSM) with regard to grain yield. There were no significant differences in grain yield among varieties or across N treatments, except relative to N0 (Tables 1 and 2). A two-year field experiment conducted by Hou *et al*.^18^ revealed that the average grain yield of rice hybrid Liangyou 3905 was *ca* 9.2 ton ha^−1^ even under optimal 165 kg N ha^−1^ and 24–27 × 10^4^ hills ha^–1^ planting density. Here, the average grain yield for inbred varieties under N1 (135 kg N ha^−1^) were 9.8 ton ha^−1^ at Jiangzhou and 8.1 ton ha^−1^ under N1 (120 kg N ha^−1^) at Hangzhou. Thus, inbred varieties achieved equal or higher grain yield than super/ordinary hybrid varieties and are relatively less dependent on exogenous nitrogen application. Huang *et al*.^15^ proposed that high planting density at reduced nitrogen application rate increases grain yield and NUE in hybrid rice varieties even under low light-intensity stress. However, our previous studies showed that super/ordinary hybrid varieties are more sensitive to shade stress than inbred rice varieties. Shade stress at flowering caused substantially higher yield losses by super/ordinary hybrid rice varieties than it did for inbred rice varieties^25,26^. Inbred rice varieties may be better suited for high planting density at reduced nitrogen application rate than super/ordinary hybrid rice varieties.

Inbred varieties can attain equal or higher grain yield than hybrid varieties under high planting density in combination with reduced nitrogen application rate, as the former showed superior sources and sinks. High planting density and low nitrogen application rate markedly increased panicle number and spikelets per panicle in inbred varieties, compared with hybrid varieties. Correlation analyses showed that N was significantly (*P* < 0.01) and positively correlated with panicle number, spikelets per panicle, biomass production at flowering, and biomass production after flowering in inbred varieties. In contrast, N was significantly (*P* < 0.01) and positively correlated only with panicle number and biomass production at flowering in hybrid rice varieties. Compared to N0, under the other N treatments evaluated here, the number of panicles increased by 22.1% and by 32.1% in the hybrid and in the inbred varieties, respectively (Tables 3 and 4). Similarly, the number of spikelets per panicle increased by 27.0% in the inbred varieties but only by 9.9% in the hybrid varieties (Tables 3 and 4). The relatively higher number of panicles and number of spikelets per panicle in inbred varieties were attributed to an increase in number of tillers at higher planting density^27^. Thus, a higher planting density compensated for the negative effect of a reduced N application rate for inbred rice but not for super hybrid rice, in which case, high yield was attributed to an increase in the number of spikelets per panicle and to a greater biomass production. These factors did not apply to ordinary hybrids or inbred cultivars^20–22^. In contrast, these advantages were not observed at high planting density combined with reduced nitrogen application rate. Hybrid and super hybrid rice cultivars often achieve high yields under optimum growing conditions, especially at high N input. Thus, hybrid and super hybrid rice may perform better than inbred rice only at high N application rate^20,28–30^. Here, hybrids did not show any advantage over inbred varieties with respect to number of panicles per square meter, number of spikelets per panicle, or biomass production at low N input rate combined with high planting density. Hybrids had higher grain weight than inbred varieties but this discrepancy did not compensate for the detrimental treatment effects of reduced N rate.

Hybrid rice is well adapted to high N fertilizer conditions and requires large amounts of N fertilizer to produce high yields. Consequently, farmers tend to apply substantial quantities of N fertilizer aiming to ensure high grain yields. However, heavy N fertilizer application may result in low NUE because of ammonia volatilization, denitrification, surface runoff, and leaching into the soil floodwater system^31,32^. Numerous improvements in N fertilizer management practice have been developed to increase NUE in rice production. High planting density at low N input rates is widely regarded as a sustainable strategy to improve NUE. Nevertheless, few studies have focused on the relative differences in NUE between inbred and hybrid varieties sown at high density and reduced nitrogen application rate.

The response of NUE to high planting density at low nitrogen input rate observed in the experiments reported herein were consistent with previously reported results^18,19,27^. Compared with high N application rate, AE, RE and PFP increased by 24.6%, 28.0% and 33.3% in inbred varieties, and by 32.2%, 29.3% and 35.0% in hybrid varieties, respectively, under low N application rate (Table 5 and Table 6). Therefore, this type of management practice effectively improved NUE in both hybrids and inbred cultivars. Moreover, inbred varieties showed higher NAE, NRE, and PFP than hybrid rice varieties across nitrogen treatments. To the best of our knowledge, this study is the first to compare NUE between inbred and hybrid rice varieties planted at high density and low N input rate. Enhanced NUE in inbred varieties was attributed to their comparatively lower N requirements for growth and yield formation than those of hybrid varieties. NUE was negatively correlated with N application rate^33^. Excessive N fertilizer application resulted in high soil residual nitrate levels^34^. Increased soil residual-nitrate may increase the risk of nitrate leaching and low NUE. High planting density at reduced nitrogen application rate enabled inbred rice varieties to achieve both high grain yield and high NUE.

## Conclusions

Our study demonstrated that a higher number of panicles and of spikelets per square meter largely explained the comparatively higher yield of inbred rice varieties cultivated under high planting density combined with reduced nitrogen input rate. Furthermore, inbred varieties showed higher nitrogen absorption efficiency, nitrogen recovery rate, and partial factor productivity than hybrids under all nitrogen treatments. Increasing planting density may compensate for the negative effects of a reduced nitrogen application rate in inbred varieties. Thus, high planting density combined with reduced nitrogen application rate is better suited for rice production if inbred, rather than hybrid varieties, is used.

## Methods

### Site description

Field experiments were conducted in the experimental farm at Yangtze University, Jingzhou, in 2017 and in Hangzhou, Zhejiang Province in 2018. Before transplanting and fertilizing, five soil cores were collected diagonally from the 0–20 cm soil layer in the rice paddy at the two sites, and basic soil properties were analyzed after Lu^35^. The soil at the Jingzhou site was calcareous alluvial with pH 6.8, 18.5 g kg^−1^ organic matter, 110.5 mg kg^−1^ alkali-hydrolysable N, 25.0 mg kg^−1^ available P, and 105.5 mg kg^−1^ available K. The soil at the Hangzhou site was a sandy loam with pH 7.0, 7.1 g kg^−1^ organic matter, 237 mg kg^−1^ alkali-hydrolyzable N, 17.1 mg kg^−1^ available P, and 139 mg kg^−1^ available K.

Urea at 50%, 20%, 20%, and 10% was applied at transplanting, tillering, panicle initiation (PI), and heading, respectively. There were two split potassium applications in the form of KCl. The rate was 40 kg K_2_O ha-1 and 50% was applied as a basal dressing and 50% was applied as broadcast at PI. Phosphorus and zinc were broadcast as basal fertilizer in the forms of calcium superphosphate at a rate of 30 kg P_2_O_5_ ha^−1^ and zinc sulfate at a rate of 5 kg ZnSO_4_ ha^−1^. Crop management followed standard cultural practices. Insects were intensively controlled with pesticides to avoid biomass and yield losses.

### Experimental design

Treatments were arranged in a split-split plot design with N treatment as the main plot, planting density as the subplot, and varieties as sub-subplot. Three replications were included each year. Each plot was 30 m^2^. Hybrid varieties Zhongzheyou8 (ZZY-8) and C-liangyouhuazhan (C-LYHZ), and inbred varieties Huanghuazhan (HHZ) and Yangdao 6 (YD-6) were grown at Hangzhou. Hybrid varieties Y-liangyou900 (Y-LY900) and Quanliangyouhuazhan (QLYSM), and inbred varieties Huanghuazhan (HHZ) and Yuenongsimiao (YNSM) were grown at Jingzhou. These varieties are extensively planted in southern China. Varietal specifications are listed in Table 7.

**Table 7:** Rice varieties used in this study.

Variety	Group	Year of release	Female parent	Male parent
Huanghuazhan (HHZ)	Inbred	2005	Huangxianzhan	Fenghuazhan
Yuenongsimiao (YNSM)	Inbred	2011	Huanghuazhan	Yuetai13
Yangdao6 (YD-6)	Inbred	1997	Yangdao4	Yan3021
Yliangyou900 (Y-LY900)	Hybrid	2015	Y58S	R900
Cliangyouhuazhan (CLYHZ)	Hybrid	2015	C815S	Huazhan
Quanliangyousimiao (QLYSM)	Hybrid	2017	Quan211S	Wushansimiao
Zhongzheyou8 (ZZY-8)	Hybrid	2006	ZhongzheA	T-8

Nitrogen application rates and planting densities for inbred and hybrid varieties at Jingzhou and Hangzhou are listed in Table 8. Pre-geminated seeds were sown in a seedbed on 10 May in 2017 and on 13 May in 2018. 25-d old seedlings were transplanted on 5 June 2017 and on 7 June 2018.

**Table 8:** Nitrogen rates (N) and planting densities (D) for inbred and hybrid rice varieties at Jingzhou(2017) and Hangzhou(2018).

Site	Variety	N (kg N ha^−1^)				D (plants m^−2^)		
		N0	N1	N2	N3	D1	D2	D3
Jingzhou	Inbred	0	135	180	225	74.0	83.2	95.2
	Hybrid	0	180	225	270	37.0	41.6	47.6
Hangzhou	Inbred	0	120	165	210	60.4	78.0	95.6
	Hybrid	0	165	195	225	27.0	36.0	45.0

### Crop management

Urea at 50%, 20%, 20%, and 10% was applied at transplanting, tillering, panicle initiation (PI), and grain filling, respectively. There were two split potassium applications using KCl at a rate of 40 kg K_2_O ha^−1^; 50% was applied as a basal dressing and 50% was applied as broadcast at PI. Phosphorus and zinc were broadcast as basal fertilizer as calcium superphosphate at a rate of 30 kg P_2_O_5_ ha^−1^, and zinc sulfate at a rate of 5 kg ZnSO_4_ ha^−1^. Crop management followed standard cultural practices. Insects were intensively controlled with pesticides to avoid biomass or yield losses.

### Sampling and measurements

At tillering, plant samples were separated into straw and leaves. Then, at flowering and maturity, plant samples were separated into straw, leaves, and panicles for dry weight determination after oven-drying to constant weight at 70 °C. At maturity, twelve hills were diagonally sampled over a 5-m^2^ harvest area on each plot. Three border lines were excluded to avoid border effects. Plants were hand-threshed after counting the panicles. Filled and unfilled spikelets were separated by submersion in tap water. Three 30 g of filled grain and 3 g of unfilled spikelet subsamples were removed to count the spikelets. Filled and unfilled spikelets were identified and separated after oven-drying to constant weight at 70 °C. Spikelets per panicle and grain filling percentage (100 × filled spikelet number / total spikelet number) were calculated. Grain yield was determined from a 5-m^2^ area in the middle of each subplot and adjusted to a moisture content of 0.14 g H_2_O g^−1^ fresh weight.

Dried leaf, stem, and panicle samples were collected at heading and straw and filled- and unfilled spikelets collected at maturity were pulverized and their N content was measured with a Skalar SAN Plus segmented flow analyzer (Skalar Inc., Breda, The Netherlands). Nitrogen content of was determined by the Kjeldahl method and N uptake in each organ was determined by multiplying its dry weight by its N content. Total N uptake at heading was estimated as the sum of leaf, stem, and panicle N uptake, and total N uptake at maturity was estimated as the sum of straw, filled-, and unfilled spikelet N-uptake.

Nitrogen agronomic efficiency (NAE) was calculated by the following formula: NAE (kg kg^–1^) = [(grain yield with N treatments - grain yield without N) / amount of N fertilizer applied].

Nitrogen recovery efficiency (NRE) was calculated by the following formula: NRE (%) = [(sum of N content in all aboveground components under N treatment - sum of N content in all aboveground components without N) / amount of N fertilizer applied].

Partial factor productivity (PFP) of applied N was calculated by the following formula: N fertilizer (PFP, kg kg^–1^) = [grain yield / amount of N fertilizer applied].

### Data analysis

Data shown are means subjected to ANOVA for significant differences subsequently separated by the least significant difference (LSD) test at 0.05 and 0.01 levels of significance. ANOVA was also performed on the N application rate, planting density, and their interactive effects. A path analysis of grain yield and yield components was also performed. The statistical software used for these analyses was SPSS v. 17.0 (IBM Corp., Armonk, NY, USA).
